# Perioperative apnea in infants with hypertrophic pyloric stenosis: A systematic review

**DOI:** 10.1111/pan.13879

**Published:** 2020-06-18

**Authors:** Fenne A. I. M. van den Bunder, Lotte van Wijk, Job B. M. van Woensel, Markus F. Stevens, L. W. Ernest van Heurn, Joep P. M. Derikx

**Affiliations:** ^1^ Department of Paediatric Surgery Emma Children's Hospital Amsterdam UMC University of Amsterdam and Vrije Universiteit Amsterdam The Netherlands; ^2^ Department of Paediatric Intensive Care Emma Children's Hospital Amsterdam UMC University of Amsterdam Amsterdam The Netherlands; ^3^ Department of Anesthesiology Amsterdam UMC University of Amsterdam Amsterdam The Netherlands

**Keywords:** apnea, IHPS, metabolic alkalosis, pyloric stenosis, pyloromyotomy, respiratory problems

## Abstract

**Background:**

Infantile hypertrophic pyloric stenosis (IHPS) leads to excessive vomiting and metabolic alkalosis, which may subsequently cause apnea. Although it is generally assumed that metabolic derangements should be corrected prior to surgery to prevent apnea, the exact incidence of perioperative apneas in infants with IHPS and the association with metabolic alkalosis are unknown. We performed this systematic review to assess the incidence of apnea in infants with IHPS and to verify the possible association between apnea and metabolic alkalosis.

**Methods:**

We searched MEDLINE, Embase, and Cochrane library to identify studies regarding infants with metabolic alkalosis, respiratory problems, and hypertrophic pyloric stenosis. We conducted a descriptive synthesis of the findings of the included studies.

**Results:**

Thirteen studies were included for analysis. Six studies described preoperative apnea, three studies described postoperative apnea, and four studies described both. All studies were of low quality or had other research questions. We found an incidence of 27% of preoperative and 0.2%‐16% of postoperative apnea, respectively. None of the studies examined the association between apnea and metabolic alkalosis in infants with IHPS.

**Conclusions:**

Infants with IHPS may have a risk to develop perioperative apnea. However, the incidence rates should be interpreted with caution because of the low quality and quantity of the studies. Therefore, further studies are required to determine the incidence of perioperative apnea in infants with IHPS. The precise underlying mechanism of apnea in these infants is still unknown, and the role of metabolic alkalosis should be further evaluated.

## 1 INTRODUCTION

Infantile hypertrophic pylorus stenosis (IHPS), the most common cause of gastric outlet obstruction in infants, is caused by hypertrophy of the distal muscular aperture of the stomach.^1^, ^2^ The incidence of IHPS is 2‐5 per 1000 live births.^1^, ^3^, ^4^, ^5^ The condition typically develops between the third and twelfth week after birth and is characterized by nonbilious, projectile vomiting.^4^

Vomiting causes loss of fluid as well as hydrogen, chloride, and potassium ions, resulting in hypochloremic hypokalemic metabolic alkalosis.^3^, ^6^ Due to fluid loss, most infants are dehydrated and may develop volume depletion and reduced glomerular filtration rate. Thus, the usual ability of the kidney to maintain a normal pH by bicarbonate excretion is impeded by chloride depletion and bicarbonate overload. Furthermore, in the distal tubule potassium is conserved at the expense of excreted hydrogen leading to increased metabolic alkalosis.^7^, ^8^ Besides renal compensation, the body is also able to compensate metabolic alkalosis by retaining carbon dioxide through hypoventilation.^7^, ^8^, ^9^ Normally, respiratory compensation is limited by decreased partial pressure of oxygen in the blood which activates the peripheral chemoreceptors and stimulates the ventilation rate.^6^, ^10^, ^11^ However, conflicting evidence exists demonstrating the occurrence of hypoventilation in reaction to metabolic alkalosis.^12^, ^13^ Furthermore, in young infants PaCO_2_ is the primary stimulus for ventilation via its influence on the pH of the cerebrospinal fluid, affecting the central chemoreceptors.^6^ Therefore, infants with IHPS may develop central apneas due to severe metabolic alkalosis.

Little is known about the incidence of perioperative apnea in infants with IHPS. Only a few case reports have been described.^7^, ^9^, ^14^, ^15^ Yet, timing of surgery is classically based on the correction of hypovolemia and metabolic derangements to prevent hypotension and respiratory complications, respectively. However, it is unknown to which extent this is necessary. Furthermore, the clinical consequences of the association between metabolic alkalosis and apnea remain unclear. If infants with IHPS have an increased risk of developing perioperative apnea, it may be necessary to monitor them to be able to identify apnea timely and to prevent further deterioration. In order to assess the incidence of apnea in infants with IHPS and to verify the possible association between apnea and metabolic alkalosis, we performed a systematic review.

## 2 METHODS

### 2.1 Protocol

A literature review was conducted according to the Preferred Reported Items for Systematic Reviews and Meta‐Analysis (PRISMA) guidelines, to examine all cases of apnea with metabolic alkalosis in infants with IHPS.^16^ The protocol was registered at PROSPERO (2018, CRD42018116707).

### 2.2 Literature search

We performed a search in PubMed, EMBASE (Ovid), and the Cochrane Library. The reference lists of the included articles were examined for additional publications, and key journals were hand searched. The search was conducted in September 2018 and repeated in May 2019. Keywords were pyloric stenosis, pyloromyotomy, metabolic alkalosis, apnea, and respiratory insufficiency. For the detailed search, see Appendix 1.

### 2.3 Eligibility criteria

All relevant studies were included regardless of study design, publication date, or language. Inclusion criteria were infants (age < 1 year) with (hypochloremic, hypokalemic) metabolic alkalosis, respiratory problems, and hypertrophic pyloric stenosis. Infants with metabolic alkalosis caused by diseases other than pyloric stenosis and infants with apnea or respiratory problems with another objectivated cause, such as congenital anomalies, idiopathic respiratory distress syndrome, infectious disease, or aspiration, were excluded from analysis.

### 2.4 Study selection and methodological quality assessment

Studies were included according to the criteria listed above. Two reviewers (FvdB and LvW) independently screened title and abstract of the studies retrieved using the search strategy. After first selection, full text was screened for final selection. Disagreements were judged by an independent third reviewer (JD). The Joanna Briggs Institute (JBI) critical appraisal checklists for case reports and case‐control studies were used to independently assess the methodological quality of the included studies.^17^ The JBI bias tools are often used for case series and based on the internationally used case report (CARE) guidelines.^18^

### 2.5 Data extraction and analysis

For all eligible studies, we extracted the patient characteristics, the pre‐ and postoperative clinical course, blood gas values, serum electrolyte values, additional medical examination, used anesthetics, and surgical procedure. Respiratory problems and corresponding interventions were recorded as well. Data were extracted by two reviewers (FvdB and LvW). If incomplete, we attempted to contact the authors to obtain additional data.

The primary endpoint of this study is to examine the incidence of perioperative apnea in infants with IHPS and secondary to verify the possible association between apnea and (hypochloremic, hypokalemic) metabolic alkalosis. We conducted a descriptive synthesis of the findings of the included studies. It was not possible to perform a meta‐analysis due to the limited number of patients.

## 3 RESULTS

### 3.1 Literature search

**FIGURE 1:**
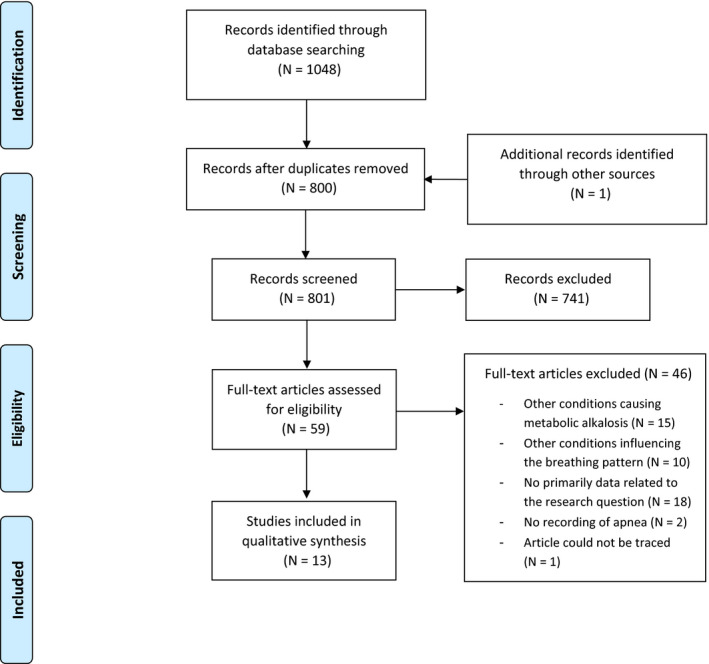
PRISMA flowchart [Colour figure can be viewed at wileyonlinelibrary.com]

The literature search initially provided 1048 potentially suitable studies. After exclusion of duplication articles, 799 studies remained, of which 741 studies were excluded subsequent to first screening. One study was added after examination of reference lists and hand searching. After full‐text screening, 46 more studies were excluded. Reasons for exclusion were other conditions causing metabolic alkalosis (N = 15 studies), other conditions influencing the breathing pattern (N = 10 studies), no primarily data related to the research question (N = 18 studies), and no recording of apneas (N = 2 studies). Furthermore, one potentially eligible study was excluded because the article could not be traced, leaving twelve eligible studies that were included. See Figure 1 for the detailed PRISMA chart.

### 3.2 Study characteristics

The thirteen eligible studies were published between 1968 and 2017, consisting of seven case reports, one case‐control study (N = 5 patients), three retrospective cohort studies, one prospective cohort study, and one randomized controlled trial (RCT) (Table 1).^7^, ^9^, ^14^, ^15^, ^19^, ^20^, ^21^, ^22^, ^23^, ^24^, ^25^, ^26^, ^27^ Except for the study by Chipps et al., the cohort studies and the RCT addressed different research questions, but described apnea as well.^23^, ^24^, ^25^, ^26^, ^27^ The other studies were case studies, in which in total 15 descriptive cases of infants with IHPS and perioperative apnea were described.^9^, ^14^, ^15^, ^19^, ^20^, ^21^, ^22^ Six studies described preoperative apnea, three studies described postoperative apnea, and four studies described both. Detailed characteristics of the included studies are summarized in Table 1.

**TABLE 1:** Details of included studies

Author(s)	Year	Country	Study design	N of cases	Respiratory problems
Cubas et al	2017	Unites States	Cohort study	n/a	Postoperative
Roben et al	2016	United States	Case report	1	Preoperative
Acker et al	2015	United States	Cohort study	n/a	Postoperative
Ein et al	2014	Canada	Cohort study	n/a	Pre‐ and postoperative
Patel et al	2013	United Kingdom	Case report	1	Preoperative
Tigges et al	2011	United States	Case report	1	Preoperative
Pappano et al	2011	United States	Case report	1	Preoperative
Galinkin et al	2001	United States	RCT	n/a	Pre‐ and postoperative
Chipps et al	1999	United States	Cohort study	30	Pre‐ and postoperative
Andropoulos et al	1993	United States	Case report	4	Pre‐ and postoperative
Abreu e Silva et al	1986	United Kingdom	Case control	5	Preoperative
Beilin et al	1985	Israel	Case report	1	Postoperative
Bennett et al	1968	United States	Case report	1	Preoperative

Note

N/a means not applicable.

### 3.3 Methodological quality

An overview of the quality assessment is presented in the Appendix S1. The overall risk of bias of the case reports is low to moderate for all case studies, except for the case report by Bennett et al, which scored high due to lack of detailed information.^9^, ^14^, ^15^, ^19^, ^20^, ^21^ Most case studies did not specify the type and volume of resuscitation fluids nor the duration of oxygen treatment or the presence or absence of hypothermia. The overall risk of bias of the case‐control study by Abreu et al^22^ is also qualified as low to moderate. Irrespective of the low to moderate risk of bias, the level of evidence of these studies is low due to the study design. The risk of bias of the study by Chipps et al was considered as high. Because the primary outcomes of all other cohort studies and the RCT were different from our intended endpoint, it was hard to define the risk of bias. Apart from Galinkin et al, none described the patient characteristics of the infants with perioperative apnea,nor the method which was used for respiratory monitoring and diagnosis of apnea.^23^, ^24^, ^25^, ^26^ Furthermore, it is unknown whether all infants of these studies received respiratory monitoring and thus whether other infants experienced (milder) apneas as well. Therefore, we indicated the risk of bias in these studies high.

### 3.4 Incidence of perioperative apnea

Five studies described the incidence of perioperative apnea in infants with IHPS, of whom only one study had the main objective to investigate whether infants undergoing pyloromyotomy have an increased risk for postoperative apnea.^27^ They conducted pre‐ and postoperative sleep studies in thirty full‐term infants undergoing pyloromyotomy. Serum electrolytes were corrected prior to surgery by using 5% dextrose in 0.45% saline with potassium chloride, and rapid sequence induction was performed by using succinylcholine, atropine, and thiopental, followed by halothane/nitrous oxide/oxygen and pancuronium for maintenance. They stated that postoperatively, infants had less central apneas and a decreased respiratory disturbance index (sum of central and obstructive apneas plus the number of hypopneic episodes per hour) compared to preoperative studies. However, 20/30 (66.7%) had abnormal preoperative sleep studies and the reported postoperative respiratory disturbance index (RDI) was at the upper end of normal (4.2; normal RDI < 5.0), suggesting that some subjects had abnormal apnea indices.^27^ In a large case series, 3 out of 791 (0.4%) infants developed perioperative apnea, one preoperatively and two postsurgery.^23^ Unfortunately, little details are given, but metabolic alkalosis of both infants who developed postoperative apnea was not adequately corrected. Other included studies described a population of infants undergoing pyloromyotomy, while comparing type of ward or anesthetic agents or evaluating the use of atropine as treatment for prolonged postoperative vomiting, respectively.^24^, ^25^, ^26^ In the retrospective cohort study of Acker et al,^24^ 5.0% of the infants developed postoperative apnea (13/259). Because the authors aimed to compare the postoperative course of infants admitted to the surgical ward postoperatively to infants admitted to the NICU for staffing or bed availability indications only, they excluded seven infants with unplanned NICU admission secondary to respiratory complications from further analysis. Type and severity of the respiratory problems of these infants were not further described. Of the remaining six infants, three developed an episode of apnea, two others suffered from hypoxia, and one patient aspirated. Galinkin et al^25^ performed a multicenter RCT comparing remifentanil‐ (N = 38) and halothane‐based anesthesia (N = 22) in infants with IHPS. All infants received a pre‐ and postoperative pneumogram to determine perioperative respiratory patterns. Apnea, defined as cessation of breathing > 15 seconds or frequent brief apneic episodes, occurred in 27% of the infants preoperatively and in 16% postoperatively. Three infants with normal preoperative recordings in the halothane group developed new onset postoperative apnea versus zero infants in the remifentanil group (23% vs 0%; *P* = .04). Five infants with abnormal preoperative recordings continued to have remarkably abnormal postoperative recordings, but in all cases subsequent recordings or follow‐up studies were completely normal. Cubas et al^26^ described two infants with episodes of postoperative apnea, requiring reintubation (2/965; 0.2%).

### 3.5 Descriptive cases

Fifteen descriptive cases from eight publications were included for separate analysis.^7^, ^9^, ^14^, ^15^, ^19^, ^20^, ^21^, ^22^ Most infants were male (80%). Age at presentation differed from 10 to approximately 60 days. All infants were born between 35 and 41 weeks of gestation. No complications during pregnancy or delivery were described, except for one infant who was born full term via cesarean section and who was small for gestational age.^19^ All infants presented with (projectile) vomiting for multiple days. Additionally, all showed signs of mild to severe dehydration such as weight loss, decreased urine output, and lethargy. Nine infants developed preoperative apnea, while five infants developed postoperative apnea and one developed both. An overview of the descriptive cases with pre‐ and postoperative apnea is shown in Tables 2 and 3, respectively.

**TABLE 2:** Overview of cases of infants with IHPS and preoperative apnea

Case	Study	Sex	Age (d)	Term/preterm	Dehydration	Additional medical examination	Laboratory values	Respiratory distress	Intervention
1	Pappano et al	F	60	Term	Severe	Rapid influenza A and RS‐virus antigens; lumbar puncture; chest X‐ray; head CT.	pH 7.48; bicarbonate 38 mmol/L; BE 24.1; pCO_2_ 68.8 mm Hg; Na 135 mmol/L; K 2.7 mmol/L; Cl 67 mmol/L	Bradypnea (12/min), SpO_2_ 88%. Shallow respiration. 3 apneic episodes, self‐resolving/responding to stimulation	1 L supplemental oxygen, bolus normal saline and iv fluids
2	Roben et al	M	10	Term	Severe	Complete blood workup and urine analysis; lumbar puncture; ECG; chest X‐ray.	Bicarbonate 30 mmol/L; Na 145 mmol/L, K 3 mmol/L; Cl 92 mmol/L	Bradypnea (10‐12/min), SpO_2_ 98%. 20 s apnea without O_2_ desaturation. In sleep, respiratory rate <10/min	Bolus 20 mL/kg normal saline. Flow‐inflating bag with 100% oxygen, high‐flow nasal cannula, intubation
3	Patel et al	M	35	Term	Severe	Urine dipstick; ECG; babygram X‐ray.	pH 7.53; bicarbonate 55 mmol/L; BE 27.8; pCO_2_ 69 mm Hg; Na 135 mmol/L; K 1.8 mmol/L; Cl 83 mmol/L	Shallow respirations (28/min), intermittent desaturations (SpO_2_ 70%‐80%). Brief, persistent apneic episodes. Poor respiratory drive	36% oxygen, intubation. 2 boluses of normal saline
4	Tigges et al	F	35	Term	Mild/moderate	Complete blood count, liver profile; abdominal X‐ray.	Bicarbonate 34.3 mmol/L; Na 130 mmol/L; K 3 mmol/L; Cl 71 mmol/L	Respiratory rate 34/min, SpO_2_ 97%. Episodes of desaturation and bradycardia associated with apnea, resolving with stimulation	Emergent intubation (FiO_2_ 0.3). 2 fluid boluses
5	Bennett et al	M	42	N/a	Severe	N/a	pH 7.1; bicarbonate 12.0 mmol/L; BE −13.0; pCO_2_ 23 mm Hg	Gasping respiration	N/a
6	Abreu et al	M	Mean 35 (range 21‐49)	N/a	Mild	N/a	pH 7.53; bicarbonate 40.6 mmol/L; BE 21.6; pCO_2_ 55.1 mm Hg; Na 138 mmol/L; K 2.4 mmol/L	>15 s attack of central apnea in both active sleep and quiet sleep. 4/5 infants had prolonged attacks of obstructive apnea	N/a
7	"	M	"	N/a	Mild	N/a	pH 7.49; bicarbonate 35.0 mmol/L; BE 11.0; pCO_2_ 44.0 mm Hg; Na 138 mmol/L; K 3.7 mmol/L; Cl 94 mmol/L	4/5 infants had prolonged attacks of obstructive apnea	N/a
8	"	M	"	N/a	Mild	N/a	pH 7.49; bicarbonate 34 mmol/L; BE 9.5; pCO_2_ 42.8 mm Hg; Na 136 mmol/L; K3.6 mmol/L; Cl 86 mmol/L	4/5 infants had prolonged attacks of obstructive apnea	N/a
9	"	M	"	N/a	Mild	N/a	pH 7.48; bicarbonate 29.3 mmol/L; BE 6.6, pCO_2_ 39.4; Na 139 mmol/K; K 4.3 mmol/L; Cl 98 mmol/L	4/5 infants had prolonged attacks of obstructive apnea	N/a
10	"	M	"	N/a	Mild	N/a	pH 7.50; bicarbonate 30.9 mmol/L; BE 8.8; pCO_2_ 41.8 mm Hg; Na 138 mmol/L; K 3.4 mmol/L; Cl 96 mmol/L	>15 s attack of central apnea in both active sleep and quiet sleep. 4/5 infants had prolonged attacks of obstructive apnea	N/a

Note

F = female/M = male; N/a means not applicable or no answer available.

**TABLE 3:** Overview of cases of infants with IHPS and postoperative apnea

Case	Study	Sex	Age (d)	Term/preterm	Dehydration	Fluid resuscitation	Anesthetic agents	Respiratory distress	Intervention
1	Andropoulos et al	M	34	Term	Mild	Rehydration for 2 h with normal saline 20 mL/kg	Atropine, thiopental, succinylcholine, nitrous oxide, halothane, pancuronium	2 episodes of apnea (15‐20 s) shortly after arrival at PACU. SpO_2_ decreased from 99% to 93% with 3 L/min nasal cannula	Tactile stimulation
2	"	M	19	Term	N/a	Ringers lactate 20 mL/kg and 14 h of rehydration.	Atropine, thiopental, succinylcholine, nitrous oxide, halothane, vecuronium	2 brief episodes of apnea (each 5‐10 s), shortly after detubation. Resolved spontaneously. 15 min later, single apneic episode (15 s) without decrease in SpO_2_ with 8 L O_2_/min	Tactile stimulation
3	"	M	11	Term	Mild	N/a	Atropine, thiopental, succinylcholine, halothane, vecuronium	Perioperative, 45 min after uncomplicated induction, brief decrease in SpO_2_ (77%) (<30 s) with lack of adequate breath sounds. 7 h postoperative 2 gasping respirations and then apneic and pulseless. Generalized seizure shortly after the arrest. 7 d postoperative 2 apneic spells with bradycardia and cyanosis (20 s)	Perioperative reintubation. Postoperative cardiopulmonary resuscitation and intubation
4	"	F	19	Term	Moderate	Fluid therapy for 10 h	Atropine, thiopental, succinylcholine, isoflurane, vecuronium	Preoperative, 3 brief episodes of apnea (<15 s) with bradycardia to 100/min. 1 h postoperative, 2 episodes of apnea (15 s) with bradycardia to 100/min, SpO_2_ decreasing from 100% to 90%	Pre‐ and postoperative caffeine citrate 10 mg/kg. Tactile stimulation
5	Beilin et al	M	15	Preterm	Severe	N/a	Nitrous oxide and halothane	2‐3 min after detubation apnea and bradycardia (80‐90/min). Episodes of apnea recurred every 2‐3 min during the following 30 min	Oxygen administration by mask and bag. Naloxone 0.02 mg (8 µg/kg) iv

Note

F = female/M = male; N/a means not applicable or no answer available.

The severity of preoperative apnea varied from spontaneous recovery or only tactile stimulation to endotracheal intubation. Infants in a case‐control study showed an increased apnea index, rate of apnea, and duration of apneas during the preoperative period in both active and quiet sleep when compared with the postoperative period or matched controls (Table 2; cases 7‐10).^22^ Furthermore, respiration rates were lower and the total duration of periodic breathing during the preoperative period was increased when compared with healthy controls. Two infants had prolonged attacks of central apneas (>15 seconds) in both active and quiet sleep, and four infants had prolonged attacks of obstructive apnea (>6 seconds) in active sleep. Prolonged apneas were no longer observed after recovery or in healthy controls. The second case presented with bradypnea (12 breaths/min) and oxygen saturation of 88% while breathing room air and showed three episodes of apnea.^14^ Cases 2, 3, and 4 required intubation due to severe respiratory problems (Table 2).^9^, ^19^, ^20^ Case 2 showed bradypnea (10‐12 breaths/min) and apnea of 20 seconds.^19^ He responded well to high‐flow nasal cannula, but when asleep his respiratory rate decreased below 10 breath/min and invasive mechanical ventilation was necessary. Cases 3 and 4 had poor respiratory drive and bradycardia associated with apnea.^9^, ^20^ Case 5 describes an infant with gasping respiration and shock due to severe hypovolemia, but any further details were lacking.^7^

We found only five descriptive cases of infants with IHPS and postoperative apnea (Table 3).^15^, ^21^ The postoperative respiratory problems occurred two‐three minutes to 7 hours after surgery. All infants underwent pyloromyotomy under general anesthesia; none received spinal anesthesia. The used anesthetic agents were atropine, thiopental, succinylcholine, nitrous oxide, halothane, and pancuronium or vecuronium. In one of the infants, isoflurane was used instead of halothane. None of the infants received opioids. Case 1 developed two episodes of apnea, each lasting 15‐20 seconds, with a decrease in oxygen saturation to 93% despite 3 L/min oxygen by nasal cannula.^15^ The second case developed three episodes of apnea lasting less than 15 seconds without desaturation.^15^ He had a temperature of 35.8℃. Both cases 1 and 2 had no further apneas and were discharged home maximum within 48 hours after surgery. In the third case, intraoperative desaturation (SpO_2_ 77%) and lack of adequate breath sounds necessitated the exchange of endotracheal tube.^15^ He had no further intraoperative complications and was extubated in the operating room, but became apneic and pulseless 7 hours postsurgery. The infant was intubated and underwent successful resuscitation, but then developed generalized seizures. He was mechanically ventilated for 3 days. Seven days later, he had another 20 seconds apneic spell with bradycardia and cyanosis and was discharged 11 days after surgery with home apnea‐bradycardia monitor because of an abnormal response to a 17% oxygen challenge during polysomnography. Repeated electroencephalograms were normal, CT scan showed mild edema, and an MRI scan 10 days after the event was normal. Case 4 experienced three brief preoperative episodes of apnea (<15 seconds) with bradycardia to 100/min and two episodes of apnea (15 seconds) postoperatively.^15^ Postoperative apneic episodes were accompanied by oxygen desaturation (90%) and were successfully treated by tactile stimulation. The infant received 10 mg/kg caffeine citrate preceding surgery and additional 10 mg/kg caffeine citrate after the postoperative apneic episode. Case 5 experienced an episode of apnea and bradycardia shortly after extubation.^23^ This episode was terminated by manual stimulation and oxygen administration, but apneas persisted every 2‐3 minute during the following 30 minutes. After naloxone injection (8 µg/kg), spontaneous breathing restored and heart rate increased, although the infant had not received any opioids. No subsequent episodes of apnea occurred.

### 3.6 Apnea and metabolic alkalosis

All described infants with preoperative apnea, including the infant with both pre‐ and postoperative apnea (Table 3; case 3), had hypochloremic metabolic alkalosis.^9^, ^14^, ^15^, ^19^, ^20^, ^22^ An exception to this was case 5 (Table 2) who presented with metabolic acidosis and shock.^7^ Median preoperative serum values of all infants with preoperative apnea are shown in Table 4. In most infants, other causes of preoperative apnea were excluded based on clinical judgment and by the use of additional diagnostics including complete blood workup, urine analysis, chest X‐ray, ECG, and cerebrospinal fluid analysis (Table 2). Abreu et al^22^ did not mention whether or not they used additional diagnostics to exclude other causes of respiratory alterations, but repeated polygraphic sleep studies after recovery were completely normal.

**TABLE 4:** Median laboratory values of infants with preoperative respiratory problems.

	N	Median [IQR]	Minimum	Maximum
pH	8	7.49 [0.04]	7.10	7.53
Base excess	8	10.25 [16.33]	−13	27.8
Bicarbonate (mmol/L)	10	34.15 [8.83]	12.0	55
pCO_2_ (mm Hg)	8	43.39 [25.40]	23	69
Potassium (mmol/L)	10	3.2 [1.23]	1.8	5.3
Sodium (mmol/L)	10	138.00 [5.5]	130	149
Chloride (mmol/L)	9	92.00 [22.00]	67	104
Urea (mmol/L)	8	4.70 [5.50]	3.0	27.1

Note

Values are number of patients per variable (N), median with [interquartile range] or minimum and maximum.

Only one of five infants with postoperative apnea had noted hypochloremic metabolic alkalosis.^15^ Exact laboratory values at presentation of infants with postoperative apnea were often not available. Most infants received intravenous fluid therapy to correct metabolic derangements and/or dehydration. It is unknown what serum concentrations were required before pyloromyotomy. Although Ein et al^23^ aimed at a chloride level of ≥90 mmol/L, one of the infants with postoperative apnea had a chloride level of 85 mmol/L before surgery. Andropoulos et al^15^ described preoperative serum electrolyte levels of sodium > 137 mmol/L, potassium > 4.7 mmol/L, chloride > 101 mmol/L, and hemoglobin > 7.2 mmol/L in their infants. Other factors, which could induce postoperative apnea as anemia, hypoglycemia, sepsis, and neurological disorders, were ruled out.

## 4 DISCUSSION

We performed this systematic review to identify the incidence of perioperative apnea in infants with IHPS and to study the association between apnea and metabolic alkalosis. We identified thirteen articles describing infants with IHPS who experienced perioperative apnea, but all studies were of low quality or had other research questions. Although it is generally assumed that metabolic derangements should be corrected prior to surgery to prevent apnea, we found only one study which primarily investigated the incidence of perioperative apnea in infants with IHPS and no studies investigating the potential association of severe metabolic alkalosis with perioperative apnea.

Since most included studies were case series, we were unable to assess the incidence of perioperative apnea in infants with IHPS. Only one relatively small study addressed the exact incidence of preoperative apnea in infants with IHPS.^25^ In this study, 27% of the infants experienced apneas before surgery. Although apneas were sometimes associated with severe desaturation, the exact consequences were unclear. Furthermore Chipps et al^27^ described that 20/30 infants had abnormal preoperative sleep studies, but it is not exactly clear what was considered as abnormal. All other studies were case series, describing severe cases of apnea which required tactile stimulation, additional oxygen, or even intubation. Based on three cohort studies with other outcome measures, we found an incidence of 0.2%‐16% of postoperative respiratory complications, mainly mild apneas.^24^, ^25^, ^26^ These outcomes on the incidence of postoperative apnea in infants with IHPS are contrary to the results of Chipps et al^27^ who stated that infants undergoing pyloromyotomy are not at risk for postoperative apnea.

All described infants with preoperative apnea, except one, had moderate to severe hypochloremic metabolic alkalosis. Several dated human and animal studies have shown compensatory hypoventilation in metabolic alkalosis.^12^, ^13^, ^28^, ^29^, ^30^ This in line with more recent reports of infants with apnea caused by severe metabolic alkalosis due to (pseudo‐) Bartter syndrome, peritoneal dialysis with bicarbonate containing solutions, and consumption of gripe water, containing an high concentration of sodium bicarbonate by a previously healthy infant.^31^, ^32^, ^33^, ^34^ In infants with postoperative apnea, it was unclear whether the infants presented with metabolic alkalosis or not and whether this may have led to postoperative apnea.^22^, ^23^ Moreover, one infant had hypothermia and one an abnormal response to 17% inspired oxygen challenge 9 days after surgery.^13^ Although the authors stated that these conditions did not contribute to the apneic episodes and that they have ruled out other contributing factors, in our view this might have played a role. In all studies, it was unclear when metabolic and electrolyte derangements were considered as corrected and infants underwent surgery. In case of insufficient correction of metabolic derangements, it could be still possible that metabolic alkalosis influenced the respiratory drive. It has been suggested that cerebrospinal fluid pH may be still elevated after rapid correction of serum electrolytes, which could continuously influence the respiratory drive.^13^ Type of anesthetic technique or anesthetic agents may have played an important role in the occurrence of postoperative apnea as well. We could not compare general anesthesia and spinal anesthesia, because all infants underwent general anesthesia. Residual effects of anesthetic agents may have caused respiratory depression. Furthermore, it is well known that opioids can produce some respiratory depression. However, remarkably, all included cases underwent opioid‐free anesthesia.

The major limitations of this systematic review are the small number of included patients and the lack of cohort studies specifically investigating the occurrence of perioperative apnea in infants with IHPS. Although we initially have used the PRISMA guidelines, we did not find enough papers to conduct a quantitative meta‐analysis and were obliged to present our results by using a narrative structure. Because of the high number of case reports, there is probably publication bias. There is most probably a strong positive bias as infants with no problems were not published, but on the other hand there might be negative publication bias as dramatic cases could have been hidden. Furthermore, there are many possible and often associated causes of perioperative apnea such as prematurity, hypothermia, and residual anesthetic drug effects, which make the interpretation of data difficult. Although we have attempted to exclude patients with conditions influencing the breathing regulation or respiratory rate, some authors did not clearly mention the patient characteristics and/or complete workup. Furthermore, the normal background incidence of apnea should be taken into account. Unfortunately, due to high risk of bias no significant statements can be made in regard to the exact incidence of perioperative apnea in infants with IHPS and whether the results of this systematic review are representative for all infants with IHPS.

In spite of its limitations, the current study suggests that infants with IHPS may have a risk to develop perioperative apnea. We found an reported incidence of 27% of preoperative and 0.2%‐16% of postoperative apnea, respectively. The majority of these apneas seems to be benign and self‐limiting. However, the incidence rates should be interpreted with caution because of the low quality and quantity of the studies. Today, with the universal availability of ultrasound, the diagnosis of IHPS is made earlier and infants may present with less severe metabolic derangements. For this reason, the incidence of perioperative apnea may be decreased. Further cohort studies are required to determine the current incidence of perioperative apnea in infants with IHPS and whether it is necessary to conduct respiratory monitoring. The precise underlying mechanism of apnea in these infants is still unknown, and the role of metabolic alkalosis should be further evaluated. Depending on this association, timing of surgery could be reassessed. In the meanwhile, the standard preoperative care with normalization of metabolic derangements by fluid rehydration should be continued with respiratory monitoring of infants with IHPS and severe metabolic alkalosis.

## CONFLICT OF INTEREST

The authors have no conflicts of interest relevant to this article to disclose.

## Supporting information

Appendix S1

## Appendix 1

### SEARCH STRATEGY

#### PubMed

##### 461 hits

("Pyloric Stenosis"[Mesh] OR "Pyloric Stenosis, Hypertrophic"[Mesh] OR "Gastric Outlet Obstruction"[Mesh] OR "Pyloromyotomy"[Mesh] OR "Alkalosis"[Mesh] OR hypertrophic pyloric stenosis[tiab] OR pylorus stenos*[tiab] OR pyloric stenos*[tiab] OR pylorostenos*[tiab] OR pyloromyotom*[tiab] OR hypokalemia*[tiab] OR hypokalaemia*[tiab] OR hypopotassemia*[tiab] OR hypochloremia[tiab] OR hypochloraemia[tiab] OR metabolic derangement*[tiab] OR alkalosis[tiab] OR metabolic alkanization[tiab] OR metabolic disturbance*[tiab] OR metabolic disorder*[tiab])

#### AND

("Infant"[Mesh] OR "Pediatrics"[Mesh] OR "Child"[Mesh] OR infan*[tiab] OR babies[tiab] OR baby[tiab] OR neonate*[tiab] OR newborn*[tiab] OR child*[tiab] OR pediatric*[tiab] OR paediatric*[tiab])

#### AND

("Respiration Disorders"[Mesh] OR apnea*[tiab] OR apnoea*[tiab] OR dyspnea*[tiab] OR hypopnea*[tiab] OR respiratory problems*[tiab] OR respiratory disorder*[tiab] OR respiratory insufficienc*[tiab] OR respiratory failure*[tiab])

Embase (Ovid)

**Table:**

#	Searches	Results
1	exp pylorus stenosis/or stomach obstruction/ or pyloromyotomy/or exp alkalosis/or (hypertrophic pyloric stenosis or pylorus stenos* or pyloric stenos* or pylorostenos* or pyloromyotom* or hypokalemia* or hypokalaemia* or hypopotassemia* or hypochloremia or hypochloraemia or metabolic derangement* or alkalosis or metabolic alkanization or metabolic disturbance* or metabolic disorder*).ti,ab,kw.	85 173
2	infant/or exp pediatrics/or child/or (infan* or babies or baby or neonate* or newborn* or child* or pediatric* or paediatric*).ti,ab,kw.	3 184 691
3	exp *breathing disorder/or (apnea* or apnoea* or dyspnea* or hypopnea* or respiratory problems* or respiratory disorder* or respiratory insufficienc* or respiratory failure*).ti,ab,kw.	267 883
4	1 and 2 and 3	637
5	limit 4 to conference abstract status	189
6	4 not 5	448

#### Cochrane Library

139 hits
