# Supplementary material for: Perioperative apnea in infants with hypertrophic pyloric stenosis: A systematic review
Source: Paediatr Anaesth. 2020 Jun 18;30(7):749–58. doi: 10.1111/pan.13879 (PMC7496757; doi:10.1111/pan.13879)
Supplement: Supplementary file 1 — Appendix S1 [file PAN-30-749-s001.docx]

**Online supplements – Quality assessment**

**Figure 1. Quality Assessment Case reports**

| Critical Appraisal Case reports | | | | | | | | |
| --- | --- | --- | --- | --- | --- | --- | --- | --- |
| Study | Items | | | | | | | |
|  | Demographic characteristics | Medical history | Clinical condition | Diagnostic test, methods and results described | Treatment procedure | Post-intervention clinical condition | Adverse events | Takeaway lessons |
| Tigges et al. | Yes | Yes | Yes | Yes | No | Yes | Unclear | Yes |
| Bennett et al. | No | No | No | No | No | No | Unclear | Yes |
| Pappano et al. | Yes | Yes | Yes | Yes | No | Yes | Unclear | Yes |
| Andropoulos et al. | No | No | No | No | Yes | Yes | Unclear | Yes |
| Roben et al. | Yes | Yes | Yes | Yes | No | Yes | Unclear | Yes |
| Patel et al. | Yes | Yes | Yes | Yes | No | Yes | Unclear | Yes |
| Beilin et al. | Yes | Yes | No | No | Yes | Yes | Unclear | Yes |

| Demographic characteristics | 71% | | | | 29% | |
| --- | --- | --- | --- | --- | --- | --- |
| Medical history | 71% | | | | 29% | |
| Clinical condition | 57% | | | 43% | | |
| Diagnostic test, methods and results described // | 57% | | | 43% | | |
| Treatment procedure | 30% | 70% | | | | |
| Post-intervention clinical condition | 86% | | | | | 14% |
| Adverse events | 100% | | | | | |
| Takeaway lessons | 100% | | | | | |
| Low risk of bias | Unclear risk of bias | | High risk of bias | | | |

| Critical Appraisal Case control study | | | | | | | | | | |
| --- | --- | --- | --- | --- | --- | --- | --- | --- | --- | --- |
| Study | Items | | | | | | | | | |
|  | Comparable groups | Matched cases and controls | Same criteria for identification | Exposure in valid, standard way | Exposure measured equally | Confounding factors identified | Strategies for confounding factors | Standard and valid assessment of outcomes | Long enough exposure period | Appropriate statistical analysis |
| Abreu da Silva et al. | Yes | Unclear | Yes | Yes | Yes | Unclear | No | Yes | Yes | Yes |

**Figure 2. Quality Assessment Case control study**

| Critical Appraisal Case control study | | | | | | | | | | | |
| --- | --- | --- | --- | --- | --- | --- | --- | --- | --- | --- | --- |
| Study | Items | | | | | | | | | | |
|  | Comparable groups | Exposure measured equally | Exposure in valid, standard way | Confounding factors identified | Strategies for confounding factors | Free of outcome at start study | valid and reliable measurement of outcomes | Long enough exposure period | Complete follow-up | Appropriate statistical analysis | Strategies to address incomplete follow-up |
| Chipps et al. | No | No | Yes | No | No | Unclear | Yes | Yes | Yes | No | n/a |

**Figure 3. Quality Assessment Prospective cohort study**

N/a means not applicable
